# Draft genome sequences of 14 *Lacticaseibacillus* spp. strains, representatives of a collection of 200 strains

**DOI:** 10.1128/MRA.00485-23

**Published:** 2023-09-01

**Authors:** Andrea Colautti, Nicolò Rossi, Carla Piazza, Giuseppe Comi, Lucilla Iacumin

**Affiliations:** 1 Department of Agricultural, Food, Environmental and Animal Science (Di4A), University of Udine, Udine, Italy; 2 Department of Mathematics, Computer Science, and Physics, University of Udine, Udine, Italy; University of Rochester School of Medicine and Dentistry, Rochester, New York, USA

**Keywords:** *Lacticaseibacillus*, WGA-LP

## Abstract

Lactobacilli have a fundamental role in the food industry as starters and probiotics, therefore, requiring special attention concerning food safety. In this work, 14 strains selected accordingly to their genetic fingerprint and physiologic characteristics are presented as representatives of a collection of 200 strains.

## ANNOUNCEMENT

Lactobacilli are ubiquitous lactic acid bacteria and are of particular interest, given their significant presence in fermented and non-fermented foods and in the human commensal microbiota. Thanks to their long history of human use and consumption and their use as probiotics, they have been generally recognized as safe (1). However, despite the numerous possible beneficial effects reported in the literature (2), several cases of infections caused by these bacteria have been reported over time in immunodeficient subjects, likely due to the presence of virulence genes and antibiotic-resistance genes (3).

As recently stated by EFSA (European Food Safety Authority), the whole-genome sequencing constitutes an adequate tool to taxonomically characterize and carry out a risk assessment by verifying the presence of health concern factors in microorganisms intentionally used in the food chain (4). Therefore, this work aimed to provide the genome sequence of highly diverse representative strains to have the opportunity to clarify some specific genetic traits depending on the origin of the strains and associated with the virulence factors in the strictly connected species *Lacticaseibacillus rhamnosus*, *Lacticaseibacillus paracasei, Lacticaseibacillus casei,* and *Lacticaseibacillus zeae* recently reclassified (5, 6). These strains were selected as representative from a collection of 200 strains that were characterized using RAPD (random amplification of polymorphic DNA), Rep-PCR (repetitive-element PCR), Sau-PCR, and MLST (multilocus sequence typing) based on stress-related genes. Taking into account all the techniques employed, the fingerprint analyses allowed to clusterize the strains on the bases of their genetic profile. One representative strain per each different genetic profile was selected for the whole-genome sequencing for a total of 14 strains (7–9). The culture collection was cryopreserved in MRS (De Man, Rogosa, and Sharpe) broth (Oxoid, Italy) and added with 20% glycerol until their use for the analysis. For the sequencing process, each freeze-dried strain was cultured in MRS broth at 30°C for 48 h. After centrifugation for 5 min at 5,000 ×  *g,* the DNA was extracted using the phenol-chloroform method (10), and genomic libraries were constructed employing the TruSeq DNA PCR-Free LT Kit (Illumina, USA) using 2.5 µg of genomic DNA, which were fragmented with a Bioruptor NGS Ultrasonicator (Diagenode, USA), followed by size evaluation using Tape Station 2200 (Agilent Technologies) (Table 1).

**TABLE 1:** Statistics of assembled genomes

GenBank accession no.	SRA accession no.	Raw reads^*a*^	Strain	Organism name	Source	Provenience^*b*^	Genome size^*a*^	Contigs^*a*^	N50^*a*^	G + C content (%)^*c*^	CDS^*a*^	Completeness (%)^*d*^
GCA_028878355.1	SRR17145328	720,624	LMG 25883	*L. paracasei*	Dairy Products	LMG	3,017,070	54	99,247	46.25	2,882	99.46
GCA_028878315.1	SRR17145327	755,994	DSM 4905	*L. paracasei*	Human	DSM	3,097,123	58	137,688	46.29	2,954	99.46
GCA_028878305.1	SRR17145323	740,902	NRRL B-456	*L. paracasei*	Unknown	ARS	3,118,403	117	102,794	46.19	2,997	99.46
GCA_028878235.1	SRR17145322	748,130	M268	*L. paracasei*	Dairy Products	POT	2,730,606	140	57,940	46.28	2640	99.39
GCA_028878245.1	SRR17145321	850,782	O14	*L. rhamnosus*	Dairy Products	POT	2,910,638	39	283,390	46.7	2,693	99.46
GCA_028878215.1	SRR17145320	737,698	UD2202	*L. zeae*	Dairy Products	UDI	3,038,780	42	179,246	47.97	2,778	99.46
GCA_028878255.1	SRR17145319	886,860	I2	*L. paracasei*	Sourdough	CAM	2,992,737	157	48,532	46.41	2,835	99.46
GCA_028878205.1	SRR17145318	1,742,298	UD1001	*L. casei*	Human	UDI	3,147,269	41	276,690	47.88	2,900	99.46
GCA_028878145.1	SRR17145317	2,788,788	N1110	*L. rhamnosus*	Human	CAM	3,068,245	84	119,405	46.57	2,848	99.46
GCA_028878115.1	SRR17145316	1,930,166	N202	*L. rhamnosus*	Human	CAM	2,882,421	59	123,389	46.57	2,699	99.46
GCA_028878345.1	SRR17217968	1,639,344	UD193	*L. rhamnosus*	Dairy Products	UDI	3,114,057	46	196,800	46.69	2,912	99.46
GCA_028878125.1	SRR17145326	788,482	Mo2	*L. rhamnosus*	Human	CAM	2,943,670	63	119,233	46.62	2,706	99.46
GCA_028878105.1	SRR17145325	782,760	TMW 1.300	*L. paracasei*	Beer	LTM	3,178,055	136	60,472	46.13	3,108	99.46
GCA_028878155.1	SRR17145324	932,552	DIALYac	*L. paracasei*	Dairy Products	UDI	3,037,719	95	125,133	46.24	2,950	99.46

^*a*^ Determined using PGAP.

^*b*^ Determined using Quast.

^*c*^ Determined using CheckM.

^*d*^ Provenience: LMG: BCCM/LMG, Belgian Co-ordinated Collections of Micro-organisms (BCCM), Belgium; DSM: DSM, Deutsche Sämmlung von Mikroorganismen und Zellkülturen, Braunschweig, Germany; ARS: ARS Culture (NRRL) Collection, U.S. Department of Agriculture, USA; POT: Scuola di Scienze Agrarie, Alimentari e Ambientali, Università degli Studi della Basilicata, Potenza, Italy; UDI: Dipartimento di Scienze degli Alimenti, Università degli studi di Udine, Udine, Italy; CAM: Dipartimento di Agricoltura, Ambiente e Alimenti, Università degli Studi del Molise, Campobasso, Italy; LTM: Lehrstuhl fürTechnische Mikrobiologie, Technische Universität München, Freising, Germany.

Library samples were loaded into a Flow Cell V3 600 cycles (Illumina, USA) according to the technical support guide. Draft genome sequencing was performed through the genomic platform consisting of a MiSeq (Illumina, UK), following the protocol of the supplier (Illumina, UK). Fastq files of the 250-bp paired-end reads obtained from targeted genome sequencing of the isolated strains were used as input for genome assemblies.

The reads were analyzed and assembled with the WGA-LP pipeline (11) using the following tools included in the pipeline with default settings. The reads were qualitatively trimmed (Phred >20), and the Illumina adapters were removed using Trimmomatic v0.39 (12), validating the results with FastQC v0.11.9 (13). The presence of any contamination was verified by Kraken2 v2.0.8-b (14). The assembly was made using SPAdes v3.15.2 (15), reordering the resulting scaffolds by aligning them with reference sequences (*L. casei* 12A NZ_CP006690.1, *L. paracasei* ATCC334 NC_008526.1, and *L. rhamnosus* GG NC_013198.1) using Mauve v2.4.0 (16). The final quality of the assemblies was then evaluated by CheckM v1.1.3 (17), SamTools v1.10 (18), and Quast v5.0.2 (19). Functional annotation was carried out on the genomes using PGAP 2022-04-14.build6021 (20).

## Data Availability

Sequences were deposited in GenBank with BioProject accession number PRJNA786620. Table 1 reports for each sample the taxonomical identification, the isolation source, GenBank accession number, sequencing and assembly statistics, and genome features of strains.
